# Sharing insect data through GBIF: novel monitoring methods, opportunities and standards

**DOI:** 10.1098/rstb.2023.0104

**Published:** 2024-06-24

**Authors:** Cecilie S. Svenningsen, Dmitry Schigel

**Affiliations:** Global Biodiversity Information Facility, Universitetsparken 15, 2100 København Ø, Denmark

**Keywords:** biological data management, open data, scientific reproducibility, scientific credit

## Abstract

Technological advancements in biological monitoring have facilitated the study of insect communities at unprecedented spatial scales. The progress allows more comprehensive coverage of the diversity within a given area while minimizing disturbance and reducing the need for extensive human labour. Compared with traditional methods, these novel technologies offer the opportunity to examine biological patterns that were previously beyond our reach. However, to address the pressing scientific inquiries of the future, data must be easily accessible, interoperable and reusable for the global research community. Biodiversity information standards and platforms provide the necessary infrastructure to standardize and share biodiversity data. This paper explores the possibilities and prerequisites of publishing insect data obtained through novel monitoring methods through GBIF, the most comprehensive global biodiversity data infrastructure. We describe the essential components of metadata standards and existing data standards for occurrence data on insects, including data extensions. By addressing the current opportunities, limitations, and future development of GBIF's publishing framework, we hope to encourage researchers to both share data and contribute to the further development of biodiversity data standards and publishing models. Wider commitments to open data initiatives will promote data interoperability and support cross-disciplinary scientific research and key policy indicators.

This article is part of the theme issue ‘Towards a toolkit for global insect biodiversity monitoring’.

## 1. Introduction

Biodiversity observations play a crucial role in our understanding of ecological systems, providing valuable insights into species distributions, community dynamics, and ecosystem functioning [1]. Observations are essential in the context of insect monitoring, given the class's unparalleled and poorly represented diversity and ecological significance. As the most diverse and abundant group of animals in the terrestrial realm, insects contribute to numerous ecological processes, such as pollination, nutrient cycling, and pest control [2,3]. Therefore, accurate and comprehensive insect monitoring is pivotal for both scientific research and policy development aimed at conserving the full range of taxonomic diversity and sustaining ecosystem services. However, to fully use insect data in research, policy and conservation, data must be findable, accessible, interoperable and reusable (FAIR) [4].

One notable platform that has emerged as a powerful tool for sharing diversity data while maintaining attribution and academic credit is the Global Biodiversity Information Facility (GBIF). GBIF's international network assembles the most comprehensive modern access point to known digital species occurrence data [5]. The platform serves as a central discovery hub for data hosted by the publisher network where researchers, policymakers and practitioners access and exchange high-quality biodiversity information [6]. Indexing, searching and filtering are possible thanks to international data standards such as Darwin Core and its extensions [7]. Cores are standardized sets of data terms that capture essential information about occurrences, events or taxonomic checklists. Extensions, on the other hand, allow the inclusion of additional specialized terms that go beyond the basic core fields. Researchers can contribute data to GBIF, significantly enhancing our understanding of diversity patterns, ecological interactions and distribution dynamics, and data published through GBIF play a vital role in policy work, providing evidence for indicators and decision-making at various scales. Moreover, GBIF allocates credit to researchers whose datasets are cited by a doi-based system [8]. This system enables onward tracking of research, as in the example of a paper by Nogué *et al*. [9], whose examination of the pollination services that insects provide to European crops was cited in the 2019 Global Assessment Report on Biodiversity and Ecosystem Services [10]. Through the paper's use of GBIF-mediated data to develop its findings on interactions between pollinators and crops, it contributed to a key global policy assessment regarding sustainable agriculture, conservation of pollinator populations, and the maintenance of crop productivity.

**Figure 1.**
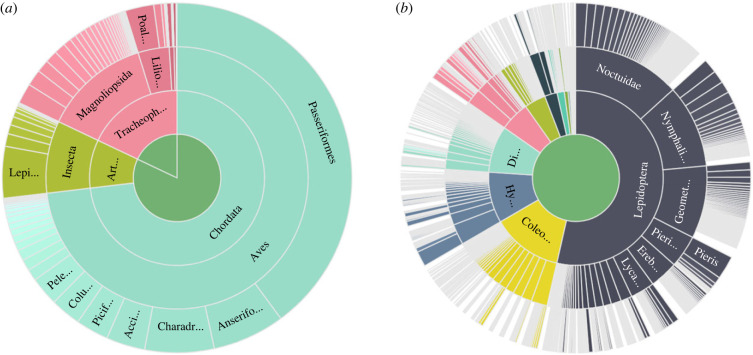
Taxonomic distribution of occurrence records in GBIF (31 January 2024), when comparing (*a*) birds (Aves, in aqua green, 73% of all records), plants (Tracheophyta ('Tracheoph...'), in dusty pink, 18% of all records) and insects (Insecta, in light green, 9% of all records). (*b*) Within class Insecta, Lepidoptera ('Lepi...' in (*a*)) observations account for more than 53% of all occurrence records.

Currently, fewer than 10% of the data available through GBIF are insect data (figure 1*a*), and the insect data are taxonomically unbalanced, with butterfly records accounting for almost half of all available records (figure 1*b*). A significant proportion of the data come from citizen science initiatives based in the United States and Western Europe (e.g. Observation.org [11], iNaturalist [12]), long-term surveys (e.g. Butterfly Conservation [13], UK Butterfly Monitoring Scheme [14]) and national databases (e.g. Artportalen (Swedish Species Observation System) [15], Finnish Biodiversity Information Facility [16]).

In recent years, technological advancements have expanded options for monitoring insect communities, enabling data collection at larger spatial scales and in finer detail than ever before [17]. Traditional methods, which rely on time-consuming and labour-intensive inventories, often fall short of capturing the true extent of insect diversity within a given area. However, the advent of novel monitoring methods, such as environmental DNA (eDNA) metabarcoding, remote sensing, computer vision and acoustics, coupled with the standardization of traditional methods [18], have dramatically expanded the potential for studying insect communities. Depending on the technology used, these new methods can cover broader geographical regions, reduce disturbance to natural habitats, and achieve more efficient data collection, while standardization of the established methods makes protocols and data available for large-scale monitoring and meta-analyses. The magnitude and pace at which insect data are being generated may now surpass the capacity of professional entomologists to effectively manage and analyse it. The loss in expertise and interpretative capabilities results in a growing need for biodiversity data infrastructures focused on traits and interactions. These infrastructures attempt to preserve and systematically organize natural history information into machine-readable formats. To be of general use, shared data need to be liberated from the context of their origins, and to some extent decontextualization [19] is achieved through data standardization. However, data make sense and are reusable if rich and adequate documentation (metadata coming from the original context) is available.

To maximize the usefulness and applicability of insect diversity data, it is imperative to ensure interoperability between different sampling methods. Interoperability enables easy combination and comparison of data from various sources, facilitating comprehensive analyses and a more holistic understanding of insect communities. Interoperability also enables researchers and policymakers to identify and address knowledge gaps needed to develop robust conservation strategies and management plans.

Biodiversity information standards developed by the Biodiversity Information Standards (TDWG) community [20] facilitate the standardization of biodiversity data, promoting interoperability and data sharing across disciplines [7]. These standards ensure consistent data structure and vocabularies while enabling integration and comparison of datasets. Important metadata on sampling methods, geospatial information, and taxonomic classifications can be consistently recorded and shared alongside the observation data.

In the following, we aim to address some of the current limitations and challenges associated with sharing data from novel insect sampling techniques with GBIF. We also address the current opportunities for sharing rich data and make concrete suggestions for relevant cores and extensions targeted at readers who may be new to biodiversity data publishing, given that researchers who engage in novel insect monitoring techniques come from a wide range of scientific disciplines.

## 2. Limitations for sharing insect data generated by novel monitoring methods

In their most simple format, occurrence data from all life on Earth can be shared, regardless of the collection or identification method. The taxonomic identity, date and location can be shared as an occurrence record at any level of detail. Most data coming from research projects, however, contain much more detailed information that can be challenging to capture with the current Darwin Core standard and extensions and sometimes fail to convey important (meta)data information that enables data to truly be FAIR. Limitations in extensive data capture are not necessarily taxon-specific, but rather sampling method- and data processing-specific.

Recognizing the need to address these challenges and accommodate diverse data types, GBIF has been working within the standards community, particularly with TDWG [20]. The ongoing development of a Unified Model [21], based on use cases and a conceptual model, aims to expand data publishing capabilities while maintaining interoperability with existing Darwin Core standards.

**Figure 2.**
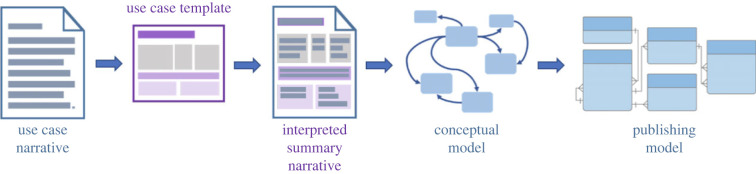
The Unified Model development process.

The approach involves soliciting narratives of use cases that present novel data publishing challenges. These narratives are transformed into interpreted summaries and used to identify the underlying concepts and relationships in the context of conceptual and practical publishing models (figure 2). Relevant use cases from the entomological community include the Global Malaise Trap Program, biotic interactions and partly eDNA metabarcoding (see ‘Case studies’ in *GBIF's new data model* [21]). However, some case studies could benefit from more direct input from researchers with a technical understanding of the novel methods, e.g. insect camera traps, remote sensing, and computer vision for species identification. For example, data relations generated by coupling traditional methods and photonic sensors [22] may be possible to capture to some degree within the GBIF's Unified Model and the relevant extensions of the international data standards; however, the complexities of identification by clustering will probably not be captured accurately as standardized fields because such data are not yet in the scope of the model.

Limitations in the current and future data schema of GBIF are not the only challenges associated with sharing data from novel insect sampling methods. Given that many of the new sampling methods are inherently novel, there is a lack of standardization associated with sampling methods and pre-processing protocols, making it difficult to define fields and terms with (controlled) values and vocabularies. Also, determining what constitutes a species unit can be highly influenced by the chosen pre-processing and annotation method. For example, the DNA-derived data extension [23] enables the sharing of DNA-derived occurrences obtained by various methods (eDNA, PCR detection etc.); however, the taxonomy associated with sequences risks being quickly outdated unless sequences are regularly re-interpreted according to updated reference databases. Reannotation is currently not a service provided by GBIF, and users of DNA-derived data would have to reannotate sequences of the dataset they use to have the most up-to-date taxonomy, or publishers would have to regularly update the taxonomy of the published dataset, every time reference databases are updated. Consequently, the reannotation of sequences would to some extent make the data download cited different from the data used, and only the sequences and the associated metadata remain stable.

Incomplete reference databases are an issue for other novel insect monitoring methods as well [24]. Reference databases for computer vision are incomplete [25], and reference databases for acoustic monitoring of insects are less complete than vertebrate reference databases [26]. However, diversity can in some cases still be examined by using the variation in the unique identifier—in the case of eDNA, the variation in DNA sequences—to infer general ecology and diversity patterns. In acoustic monitoring, family-level identification can be obtained and used for diversity analysis [17]. As most insect species remain to be described, it is expected that reprocessing and reannotation would be required for many of the novel methods to ensure data are FAIR, which currently is the responsibility of the data publishers and end users.

## 3. Current opportunities and solutions to share insect diversity data: standards, extensions and dataset examples

With the growing importance of open data and the need for collaborative efforts in biodiversity research, it is crucial to explore current opportunities and solutions to share data derived from novel monitoring methods. Here, we will discuss some of the ways in which insect data sampled with novel techniques currently can be shared with GBIF, along with examples of datasets published in GBIF collected by various novel monitoring technologies.

### (a) Sharing biodiversity data with GBIF

Researchers can contribute insect data to GBIF through GBIF-endorsed publishing organizations [27] and apply data cores and extensions to their datasets, tailored to different data types.

When publishing biodiversity data in GBIF, researchers can map their data to the appropriate cores and use relevant extensions to provide comprehensive and contextual information. The choice of core and extensions depends on the specific data type and sampling technique employed. The following covers some examples of relevant presently available cores and extensions for sharing data from novel insect monitoring techniques:

1. Occurrence core: The Occurrence core can be used to record individual organisms, observed or collected independently from each other. It includes essential information such as taxonomic identification, location and date of the observation. Insect data collected using novel sampling technologies can be mapped to this core by providing accurate taxonomic identification, reliable temporal information and precise spatial coordinates.
2. Event core: The Event core is designed to capture data associated with a specific sampling event or activity. In the context of novel sampling technologies, such as camera traps or acoustic monitoring, researchers can use the Event core to document details such as camera setup, sampling protocols, and specific monitoring locations. This core, through its focus on the spatiotemporal and sampling effort aspects of a study design, helps to provide crucial contextual information for interpreting the data on insect diversity and abundance.
3. Media extensions: Media extensions allow researchers to include various types of media files, such as images, audio recordings, or videos, alongside occurrence records. This is particularly useful when publishing insect diversity data derived from camera traps, acoustics or remote sensing. Researchers can link media files to individual occurrence records or event records, providing visual or auditory evidence to support their observations and enriching the dataset. Provision of primary media alongside its data and biological interpretation allows re-interpretations (such as re-identification) using improved reference libraries.
4. DNA-derived data extension: For DNA-derived data, GBIF has developed the DNA-derived data extension [23]. This extension enables researchers to include DNA-specific information, such as DNA sequences, genetic markers used, or laboratory protocols followed. By mapping DNA-derived data to this extension, researchers can ensure the appropriate integration of their datasets with existing DNA repositories and comply with relevant DNA data standards. The use of this extension does not duplicate services of DNA archives and repositories but ensures that DNA-derived detections and quantifications are interoperable as records of species.

By applying the appropriate cores and extensions to their datasets, researchers can enhance the discoverability, interoperability and reusability of their insect diversity datasets in GBIF. This ensures that valuable information derived from novel sampling technologies is effectively shared and integrated into broader biodiversity research efforts. For a complete list of cores and extensions used in GBIF, see https://rs.gbif.org/extensions.html.

### (b) Examples of current ways to publish data to GBIF using novel monitoring techniques

While datasets on insect occurrences derived from novel monitoring methods remain very scarce, some publishers have already shared data through GBIF obtained using novel monitoring techniques. In most cases, the datasets concern other focal taxon groups than insects. Still, the examples provided here should to a large extent cover the same needs for cores, extensions and standards required to publish datasets derived from novel insect monitoring methods.

#### (i) Acoustic monitoring

Acoustic monitoring examples are available through GBIF that may provide a template for new data publishers. For example, Xeno-canto Foundation for Nature Sounds publishes insect acoustic occurrence records as part of their greater than 600 000 records dataset [28]. The occurrence dataset contains oscillograms, spectrograms and recordings shared through the media extension.

#### (ii) DNA metabarcoding

DNA metabarcoding datasets can be published to GBIF with the occurrence core and publishers can apply the DNA-derived data extension to increase the richness of their datasets, e.g. by sharing sequences and relevant metadata from laboratory protocols and bioinformatics pipelines [23]. However, several datasets are published without the use of the extension and/or by using the event core instead, e.g. the dataset *National insect monitoring in Norway* [29]. GBIF's new data model [21] will make it possible to publish event core archives and apply the DNA-derived data extension since the event core in many cases is more immediately applicable for the original data.

#### (iii) Computer vision and camera trap data

Whether identification of taxonomic groups is acquired through manual, visual or AI identification, the publication procedures are the same when sharing data with GBIF, with minor differences in relevant fields and field values. Computer vision can be applied to a diverse range of images, and for biodiversity data, it is applied to everything from monitoring data from camera traps to digitized specimens from natural history museum collections. Computer vision can be the first attempt at identification of an observation in a citizen science platform, e.g. iNaturalist, but only research-grade observations (community-vetted) are published to GBIF, through iNaturalist [12].

There are challenges associated with publishing camera trap data to GBIF, and with no current framework or standard, the observations are shared in a variety of ways. Some datasets are published without images documenting the occurrences [30], which in some cases may be due to image hosting limitations. In other cases, one image is shared out of a sequence, as evidence of the occurrence [31].

## 4. Challenges associated with novel insect monitoring methods

Novel monitoring technologies may contribute to an increased geographical coverage of underrepresented regions, such as the tropics [32]; and it can be expected that monitoring and detection technologies will find their way to the lower-capacity parts of the world sooner than corresponding entomological expertise [33]. However, integrated approaches are needed to properly describe and understand [34] the many new species that will be revealed by an increased global coverage of insect diversity. For example, a combination of citizen science, computer vision and AI identification could be used to increase knowledge of insect diversity [35] in protected areas designated mostly on data on vertebrate and plant diversity [36]. Moving intensive insect monitoring to poorly known areas with many undescribed species will require interpretation and quantification of species hypotheses (provisional taxa and global operational taxonomic units), such as Barcode Index Numbers (BINs) in the case of eDNA [37].

Despite the potential benefits, it is important to recognize that use of novel monitoring methods cannot overcome the current limitations of GBIF-mediated insect data on its own. Implementation challenges, cost considerations and technical requirements can limit the widespread adoption of these techniques. If the techniques are mainly used in the better-described regions of the world, the spatial and taxonomic gap may widen as an effect.

Moreover, challenges related to data quality and data management practices need to be addressed to ensure the reliability and usability of insect diversity data obtained through novel monitoring methods. Standardization of data collection protocols, metadata documentation, and data curation processes is crucial for maximizing the potential of innovative approaches and promoting data sharing across research communities. Many of the novel technologies produce large amounts of complex (raw) data, e.g. camera traps, computer vision and eDNA, which can be challenging to host long-term and share through available data standards. However, as data hosting is the responsibility of the GBIF publishers, the evidence of an occurrence or event is currently defined by each individual dataset publisher. Ideally, the scientific community should engage in developing standards to allow data to be FAIR since extensive metadata documentation alone does not allow interoperability and reuse.

A specific set of challenges is, in fact, not related to data generation or data management technologies, but to data culture and efforts. The efforts necessary to share data in the FAIR way are higher than in the still widespread drop-and-forget culture of open data, where the use of supplementary materials and data repositories is driven by the need to satisfy minimum (and often externally introduced) requirements for data sharing, and not by the genuine desire of the researchers or organizational policies to enable data reuse. The minimum standard approach attempts to balance meaningful, interoperable data with ease of sharing [38,39]. The world seems to be moving towards FAIR data as a norm, but the landscape of attitudes and practices remains patchy.

To fully make the most of the potential of novel monitoring methods, collaborative efforts are needed. Partnerships between researchers, data aggregators, and data standards initiatives are necessary to develop suitable data standards and practices that can accommodate the unique characteristics of novel monitoring data and enhance the integration of these valuable datasets into global biodiversity databases. GBIF has promoted such collaborations through the Biodiversity Information for Development (BID) and Biodiversity Information Fund for Asia (BIFA) initiatives, for example through a call to mobilize DNA-derived data [40]. In addition, alignment of data practices can be implemented through coordination among the journals and publishers. By fostering these collaborations, data sharing can be enhanced, contributing to a more comprehensive understanding of insect communities and their dynamics.

## 5. Future potential for sharing insect monitoring data derived from novel sampling technologies

Novel sampling and sample processing techniques may in the future help lessen taxonomic and geographical gaps, by generating diversity data for areas that were not easily accessible by traditional methods [41], and therefore could contain unknown species for science [42]. Similarly to eDNA and metabarcoding surveys, and differently from citizen science heavily influenced by human sentiments, sampling and monitoring instruments are free from opinions and preferences, providing an additional line of hope toward more taxonomically neutral, and biologically adequate, data pools.

The taxonomic information in occurrence records in GBIF is often based on specimen identifications carried out by various contributors, including researchers, citizen scientists and data aggregators. While efforts are made to ensure accurate identifications, inconsistencies and errors in taxonomic assignments can occur. These inconsistencies may arise owing to variations in expertise, different taxonomic concepts or revisions, and difficulties in accurately identifying certain insect groups. Species units obtained by novel methods can serve as a stable, digital ID that allows reannotation as new species are identified and reference databases are becoming more complete.

As GBIF continues to grow in terms of participation, aggregated data, and citations, there is a recognized need to expand the data model to accommodate more varied data types, including data from novel insect monitoring methods. One of the challenges identified in the current model is the oversimplification of data, leading to a loss of data richness and limitations in adhering to FAIR principles. The current model is based on Darwin Core [7] and has been successful in promoting data sharing owing to its simplicity. However, the limitation of one core set of records with limited applicable extensions has in some cases resulted in simplifying otherwise rich datasets.

The Unified data model shifts the focus from individual occurrences to recording events, allowing more comprehensive and context-rich data representation. By treating each sampling event as a distinct record, the new data model captures important metadata associated with the event, such as sampling protocols, environmental conditions, and specimen handling procedures. The transition to an event-based approach improves data quality, facilitates interoperability, and supports more nuanced analyses of biodiversity. By expanding the data model and incorporating the Unified Model, GBIF aims to enhance data richness, promote interoperability, and provide a more sophisticated ecosystem for data mobilization and use in biodiversity research. At the same time, GBIF strives to flatten the learning curve and reduce the data publishing threshold by moving closer to accommodating data holders' native ways of managing data.

Another promising future addition to the Darwin Core standard, highly relevant for the insect monitoring community, is the work on the Humboldt extension, initiated by Guralnick with co-authors [43], which has now been ratified. The Humboldt extension to Darwin Core adds over 50 terms capturing sampling effort, sampling size, methods, taxonomic targets and other data essential for adequate interpretation and analysis of quantitative and presence–absence data at large scales. The incorporation will enable the standardization of data from monitoring projects that were previously only possible to capture as metadata.

## 6. Conclusion

Biodiversity observations and the sharing of insect diversity data through GBIF are crucial for advancing scientific understanding and informing evidence-based policies for insect conservation and ecosystem management. It is imperative that the scientific community around novel insect monitoring techniques engages in the development of data standards and data model expansion to ensure interoperability of the vast amount of data being produced. Interoperability between different sampling methods, techniques and technologies, and adherence to standardized data protocols, are essential for achieving comprehensive and comparable insect diversity data. Embracing FAIR data principles ensures transparency, collaboration and innovation in insect monitoring and facilitates the engagement of diverse stakeholders in conservation efforts. By promoting and following these principles, we can enhance our knowledge of insect communities and drive effective conservation actions to safeguard global biodiversity.

## Data Availability

This article has no additional data.
